# Implications of circular transcripts in DM1 pathomechanism

**DOI:** 10.1016/j.omtn.2025.102661

**Published:** 2025-08-13

**Authors:** Marzena Wojciechowska, Piotr Kozlowski

**Affiliations:** 1 Department of Rare Diseases, Institute of Bioorganic Chemistry, Polish Academy of Sciences, Poznan, Poland; 2 Department of Molecular Genetics, Institute of Bioorganic Chemistry, Polish Academy of Sciences, Poznan, Poland

## Main text

**Figure 1:**
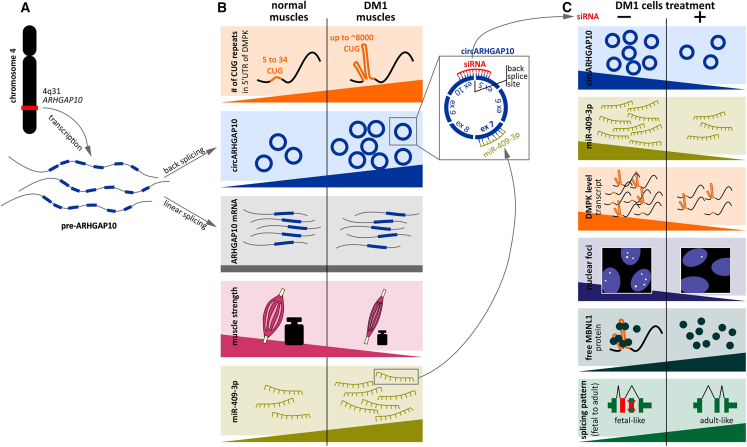
The role of circRNAs in DM1 and the potential therapeutic effect of circARHGAP10 downregulation (A) Transcription of ARHGAP10 is correlated with forward and back splicing, resulting in the generation of linear and circular transcripts, respectively. (B) Comparison of characteristic features between normal and DM1-affected skeletal muscles. (C) Treatment with siRNA targeting circARHGAP10 reverses DM1-associated molecular biomarkers.

Recently, it has been demonstrated that circular RNAs (circRNAs) are globally upregulated in myotonic dystrophy type 1 (DM1) and their levels correlate with the disease severity (Figure 1).^1^^,^^2^^,^^3^^,^^4^^,^^5^ However, the specific role of circRNAs in the pathomechanism of DM1 remains largely unknown. In a recent issue of *Molecular Therapy Nucleic Acids*, Baci and colleagues report a novel discovery titled “*circARHGAP10 as a candidate biomarker and therapeutic target in myotonic dystrophy type* *1*.”^6^ This study explores the functional relevance of specific circRNAs in DM1 and proposes a new molecular mechanism underlying DM1 pathogenesis involving circARHGAP10 and its interaction with miR-409-3p.

DM1 is a rare neuromuscular genetic disorder with no cure or effective treatment to halt or reverse its progression. In patients, the CTG repeat tract in the 3′ UTR of the *dystrophia myotonica protein kinase* (*DMPK*) gene can exceed several hundred repeats, whereas unaffected individuals typically have fewer than 10 repeats (Figure 1).^7^ The pathogenic mechanism of the mutant DMPK allele (CUG^exp^ RNA) involves a toxic *gain-of-function*, characterized by nuclear accumulation of the expanded transcript and its aberrant interactions with splicing factors from the muscleblind-like (MBNL) protein family. This leads to the misregulation of alternative splicing and expression of embryonic isoforms of numerous genes in adult tissues. However, spliceopathy represents only one aspect of the broader RNA metabolism abnormalities that define DM1 pathogenesis. For the past two decades, most research in this area has focused on the causes and consequences of alternative exon usage, with MBNL proteins widely recognized as central players in this process. Yet, recent studies investigating circRNA dysregulation in tissues from patients with DM1 have not established a direct link between MBNL functional deficiency and circRNA upregulation. In light of current knowledge, the discovery of dysregulated circRNAs broadens the spectrum of molecular abnormalities potentially contributing to DM1 onset, progression, and treatment strategies.

Baci and colleagues demonstrate that circRNA dysregulation, which features samples of a variety of skeletal muscles—including the *tibialis anterior*, *quadriceps femoris*, and *biceps brachii*—from adult patients with DM1, is also present in individuals with early-onset congenital myotonic dystrophy, the most severe form of the disease. Furthermore, by analyzing datasets from patients with other myopathic conditions such as sarcopenia and limb girdle muscular dystrophy R12, the authors show that circRNA upregulation is a DM1-specific phenomenon, rather than a general feature shared across muscle-wasting disorders or myopathies. A similar conclusion regarding the specificity of circRNA upregulation to DM1 emerges from analyses of neuronal tissues from Huntington’s disease (HD) and amyotrophic lateral sclerosis (ALS). Despite their distinct genetic backgrounds, both disorders are characterized by pronounced neurological deficits and aberrant alternative splicing. However, studies in HD and ALS mouse models did not report a global increase in circRNA levels.^8^^,^^9^ Instead, diminished production of circRNAs was observed in neural progenitor cells and the striatum in HD models, as well as in the spinal cord in ALS models. Consistent with these findings, circRNA levels also tend to decline in the central nervous system of patients with sporadic ALS.

The study centers on circARHGAP10, which exhibits a high circular-to-linear ratio and the strongest discriminatory power between patients with DM1 and healthy individuals. Notably, circARHGAP10 levels in DM1 show a positive correlation with CTG repeat length, and a negative correlation with skeletal muscle strength (Figure 1). No such associations are observed for the expression levels of the linear ARHGAP10 transcript, suggesting that the regulation of the circRNA is distinct and independent of its linear counterpart. To explore the functional role of circARHGAP10, Baci and colleagues perform *loss-of-function* experiments by silencing circARHGAP10 using siRNAs in both proliferating and differentiated DM1 myogenic cells. This intervention outcomes in a range of molecular effects impacting established DM1 biomarkers, including altered DMPK mRNA expression, reduced nuclear CUG RNA foci, increased availability of MBNL1, and changes in aberrant alternative splicing. These findings provide compelling evidence for the involvement of upregulated circARHGAP10 in the pathomechanism of DM1. Of particular interest, silencing circARHGAP10 reduces in total DMPK mRNA levels in both DM1 and control cells (Figure 1). Furthermore, this decrease is observed in both nuclear and cytoplasmic RNA fractions, suggesting a global downregulation of DMPK transcripts across cellular compartments. These findings imply that the regulatory influence of circARHGAP10 on *DMPK* expression is not allele-specific but may involve a broader post-transcriptional mechanism. This result is of particular importance, as DM1 is caused by an abnormal expansion of a (CTG)n trinucleotide repeat in the *DMPK* gene, and downregulation of its mRNA levels represents a promising therapeutic strategy. A direct consequence of this reduction is a decrease in the quantity and quality of CUG^exp^ RNA foci in DM1 cells (marking phenotype of DM1). Using fluorescence microscopy, the authors observe a significant increase in the proportion of nuclei lacking RNA foci, along with a reduction in the number, area, and intensity of CUG foci following circARHGAP10 knockdown. Notably, these effects are not replicated by silencing the linear ARHGAP10 transcript. An additional aspect of the *loss-of-function* experiments involves examining MBNL1 protein sequestration within nuclear RNA foci and associated splicing abnormalities. Through quantitative RNA fluorescence *in situ* hybridization combined with MBNL1 immunofluorescence in DM1 myogenic cells, they demonstrate a significant reduction in the number and intensity of MBNL1-positive nuclear foci per nucleus, indicating diminished sequestration. Moreover, circARHGAP10 silencing leads to a significant increase in overall MBNL1 protein levels, suggesting enhanced protein availability. Crucially, this increase in bioavailable MBNL1 is accompanied by a partial correction of splicing defects in several well-characterized MBNL1-dependent targets mis-spliced in DM1 skeletal muscle. Collectively, these findings demonstrate that circARHGAP10 silencing alleviates several key molecular features of DM1 pathology and may offer a novel therapeutic avenue.

Finally, the authors demonstrate that circARHGAP10 may function through its interaction with miR-409-3p (Figure 1). Both molecules are upregulated in muscle biopsies from patients with DM1, and siRNA-mediated silencing of circARHGAP10 in DM1 cells results in decreased miR-409-3p levels, supporting their functional interaction and possible co-regulation*. In vitro* validation of this interaction, using RNA pull-down assays and analyses of RNA association with the RNA-induced silencing complex, revealed enrichment of both circARHGAP10 and miR-409-3p in DM1 cell lysates. To further investigate this interaction, the authors assess whether an increase of miR-409-3p could reverse the molecular effects of circARHGAP10 knockdown—namely, the downregulation of DMPK mRNA, reduction in nuclear RNA foci, and partial correction of alternative splicing. Interestingly, co-transfection of DM1 cells with both si-circARHGAP10 and a miR-409-3p mimic fails to replicate the full extent of these changes, suggesting that the pathogenic effects mediated by circARHGAP10 are, at least in part, dependent on its interaction with miR-409-3p.

While this study marks an important advance in understanding the functional roles of circRNAs and their mechanistic involvement in DM1 pathogenesis, it also raises several questions that warrant further investigation. A key area for future research is the elucidation of the broader regulatory network involving circARHGAP10 and miR-409-3p, which may reveal additional modulators and pathways contributing to DM1 pathology. Moreover, such investigations may clarify whether the dysregulated biogenesis of circRNAs is a secondary consequence of mutant DMPK expression or a primary driver of the DM1 disease program.

## Acknowledgments

The authors are funded by the Polish National Science Centre (2019/33/B/NZ5/02473, 2020/39/B/NZ3/01811, and 2021/43/O/NZ1/01590 to M.W.).

## Declaration of interests

P.K. is an associate editor at *Molecular Therapy Nucleic Acids*.
